# CD147 increases mucus secretion induced by cigarette smoke in COPD

**DOI:** 10.1186/s12890-019-0791-0

**Published:** 2019-02-06

**Authors:** Qiao Yu, Danhui Yang, Xi Chen, Qiong Chen

**Affiliations:** 1 Department of Gerontology and Respirology, Xiangya Hospital of Central South University, Changsha, 410008 Hunan China; 2 Department of Respiratory Medicine, The Second Xiangya Hospital of Central South University, Changsha, 410011 Hunan China; 3 Department of Respiratory Medicine, Xiangya of Central South University, Changsha, 410008 Hunan China

**Keywords:** CD147, Mucus, MUC5AC, Cigarette smoke extract, COPD, MMP9, p38 MAPK

## Abstract

**Background:**

CD147 is expressed in many tissues and is involved in many inflammatory diseases. Emerging evidence suggests that the overproduction of mucus is a malignant factor in chronic obstructive pulmonary disease (COPD), which results in severe airway obstruction and repeated airway infections. However, it is still unclear whether CD147 is involved in mucus production in COPD.

**Methods:**

We determined the expression levels of CD147 and MUC5AC by immunohistochemistry in 42 human lung specimens from three groups (non-smokers without COPD, smokers without COPD and smokers with COPD). For the in vitro experiment, human bronchial epithelial (HBE) cells were treated with cigarette smoke (CS) extract to establish a mucus secretion model; then, CD147 and MUC5AC production were detected by RT-PCR, Western blotting and ELISA. To determine how CD147 is involved in MUC5AC secretion, HBE cells were transfected with small interfering RNA to silence CD147 and pretreated with inhibitors of MMP9 and p38 MAPK, which are common signaling molecules involved in MUC5AC secretion; then, MUC5AC expression was evaluated.

**Results:**

Compared with the expression levels in the non-smokers and smokers without COPD, CD147 and MUC5AC expression levels were higher in the smokers with COPD. In the in vitro experiment, CD147 and MUC5AC expression levels were significantly increased after CS extract incubation compared with those after no treatment. Silencing CD147 by siRNA decreased the CS extract-induced MUC5AC secretion and MMP9 and phosphorylated p38 MAPK production. In addition, inhibiting MMP9 or p38 MAPK decreased the CS extract-induced MUC5AC secretion.

**Conclusions:**

In lung specimens, CD147 and MUC5AC expression levels were increased in COPD patients. Increased CD147 levels induced by CS extract could stimulate MUC5AC secretion through the MMP9 and p38 MAPK signaling pathway in HBE cells. Therefore, the regulation of CD147 could be a promising target for mucus hypersecretion in COPD.

**Electronic supplementary material:**

The online version of this article (10.1186/s12890-019-0791-0) contains supplementary material, which is available to authorized users.

## Background

COPD is a smoking-related disease that is characterized by persistent respiratory symptoms and airflow limitations due to airway abnormalities typically caused by significant exposure to noxious particles or gases [1]. Mucus overproduction is one of the most frequent symptoms [2]. Excessive mucus synthesis results in airflow obstruction, pulmonary ventilation dysfunction and reduced cilia removal of mucus in COPD patients [1–4]. It is important to elucidate the mechanism of mucus secretion, since the targeted treatment of pathologic airway mucus not only improves the symptoms of cough and dyspnea but also decreases the frequency of disease-related exacerbation.

Cigarette smoke, the main exposure risk for COPD, impairs the capability of cilia to clear mucus; several cigarette components contribute to goblet hyperplasia and mucus secretion, and the lung becomes mucus-rich and inflamed after many years of cigarette smoke exposure [5]. The major macromolecular components of mucus are mucin glycoproteins [3]. To date, 13 human MUC genes have been found in the airway. Among them, MUC5AC is implicated in the pathogenesis of COPD as a cause of mucus airway obstruction [6–8]. MUC5AC is upregulated transcriptionally by many environmental factors, including cigarette smoke, and multiple signaling molecules, including matrix metalloproteinase 9 (MMP9), mitogen-activated protein kinase (MAPK), and epidermal growth factor receptor (EGFR), are involved in MUC5AC production [9–13]. Nevertheless, whether factors upstream of these signaling molecules participate in MUC5AC production in COPD remains unknown.

CD147 (basigin, EMMPRIN) is a widely expressed plasma membrane protein that has been implicated in a number of pathological and physiological processes; it is best known for its ability as an MMP inducer, and recent studies showed that CD147 also modulates the phosphorylation of p38 MAPK [14–17]. What is more, previous studies have demonstrated the role of CD147 in regulating inflammatory responses in a variety of diseases, including lung inflammation [18–20]. Berg et al. found that serum levels of CD147 were high in COPD patients [21]. Since MMP9 and p38 MAPK are indispensable signaling molecules in the regulation of MUC5AC secretion, we hypothesize that CD147 may take part in MUC5AC production in COPD through these signaling molecules.

In this study, we assessed the expression of CD147 and MUC5AC in lung tissues from smokers with COPD and controls. In an in vitro experiment, cigarette smoke (CS) extract was used to induce mucus hypersecretion in human bronchial epithelial cells; then, we assessed the role of CD147 in MUC5AC production and the possible molecular mechanism.

## Materials and methods

### Subjects

Forty-two subjects were recruited for this study, including 14 non-smokers without COPD, 14 smokers without COPD, and 14 smokers with COPD. The inclusion criteria were as follows: All subjects underwent pulmonary nodule resection. A diagnosis of COPD was made according to the guidelines of the Global Initiative for Chronic Obstructive Lung Disease [1]. COPD patients in our study were defined as having a phenotype of chronic bronchitis and had a history of exposure to cigarette smoke. All patients took a pulmonary function test, and forced expiratory volume in 1 s (FEV1)/forced vital capacity (FVC) < 0.70 was used to confirm the airflow limitation to diagnose COPD. All patients were subjected to serum protein electrophoresis tests, and there were no subjects with alpha-1 antitrypsin deficiency. Patients had no history of exposure to occupational dust or chemicals or indoor or outdoor air pollution. The exclusion criteria were as follows: Patients with comorbidities, including interstitial lung disease, heart failure, asthma, and neuromuscular disease, were excluded. None of the subjects had suffered from any respiratory tract infection or received any glucocorticoids or antibiotics during the month preceding the study. All lung samples were obtained from Xiangya Hospital of Central South University (Changsha, China). For each specimen, two tissue blocks (sample size 15–25 mm) were taken from the sub-pleural parenchyma of the lobe obtained in surgery, and they were from at least 5 cm away from the margin of the diseased regions. All participants signed informed consent, and this study was performed with the approval of the Ethics Committee of Xiangya Hospital.

### Histology and immunohistochemistry (IHC)

Samples of formalin-fixed lung tissue were embedded in paraffin, sectioned (4 μm) and stained with hematoxylin and eosin (HE) for morphometric measurement or with Alcian blue-periodic acid Schiff stain to assess the mucin glycoproteins in the epithelium. The tissue sections were deparaffinized and stained with mouse anti-human CD147 (1:100, Abcam) and mouse anti-human MUC5AC (1:200, Abcam) primary antibodies overnight at 4 °C; then, the sections were incubated with a horseradish peroxidase (HRP)-conjugated anti-mouse secondary antibody (1:1000, Abcam), and the color was developed using diaminobenzidine. In the negative controls, the primary antibody was substituted with phosphate-buffered saline (PBS). Immunochemistry staining of the lung tissue sections was evaluated at 200-fold magnification in four random fields per section. The mean staining intensity values of CD147 and MUC5AC in the airway epithelium were analyzed using Image-Pro Plus 7.0 software.

### Preparation of cigarette smoke (CS) extract

CS extract was prepared in a manner similar to that in a previous study [22]. CS extract (100%) was prepared by bubbling smoke from two cigarettes in 10 ml of serum-free RPMI1640 media at a rate of a half cigarette/min. The pH of the CS extract was adjusted to 7.4, the optical density was determined (0.783 ± 0.03), and the CS extract was sterile-filtered through a 0.22-μm filter. The CS extract was always prepared fresh on the day of the experiment.

### Cell culture and treatment

HBE cells are immortalized human bronchial epithelial cells that were purchased from Fuxiang Biotechnology Co., Ltd. (Shanghai, China). The HBE cells were propagated in RPMI 1640 (Gibco) supplemented with 10% FBS, 100 U/ml penicillin, and 100 μg/ml streptomycin in a 37 °C 5% CO_2_ incubator. After serum starvation for 12 h, the cells were treated with CS extract at different concentrations (0, 2, 5, 10, 15, and 20%) for 24 h or with 10% CS extract for different durations (0 h, 6 h, 12 h, 24 h, and 36 h). Before the subsequent tests, a cell counting kit 8 (CCK8, from Engreen) assay was used to evaluate cell proliferation. To investigate the signaling cascade involved in the CS-induced MUC5AC secretion, cells were pretreated with 10 μM SB-3CT (MMP9 inhibitor, Sigma) or 10 μM SB203580 (p38 MAPK inhibitor, Cell Signaling Technology) for 1 h. Following a 24-h incubation in culture medium containing 10% CS, the cells and culture supernatants were harvested for further analysis. HBE cells were transfected with CD147 small interfering RNA (RiboBio) using Lipofectamine 3000 (Invitrogen) to silence CD147; then, the cells were incubated for 2–3 d, and the transfection efficacy was determined. Finally, the cells were treated with CS extract (10%) for 24 h.

### Enzyme-linked immunosorbent assay (ELISA)

Cell culture supernatants were collected and used to assay the total protein concentrations. MUC5AC protein levels were measured following protocols provided by the ELISA kit manufacturer (Sangon, Shanghai). Protein was extracted as soon as possible after specimen collection. The standard was diluted; then, the samples, standards and blank were added to the wells of the plate and incubated for 1 h at 37 °C. The liquid was discarded; then, the plate was washed 5 times and patted dry. Chromogenic reaction reagent was added and incubated in the dark for 15 min at 37 °C. Finally, stop solution was added, and the absorbance at 450 nm was measured within 10 min.

### Real-time polymerase chain reaction (RT-PCR) analysis

Total RNA was extracted from the HBE cells in each group using TRIZOL (Invitrogen). cDNA was generated using a Transcriptor First Strand cDNA Kit (Roche) in accordance with the manufacturer’s protocol. Quantitative RT-PCR was performed using a StepOnePlus PCR system (Applied Biosystems) and gene expression assays. The PCR primers (Sangon) used for MUC5AC were 5’-TACTCGCTCGAGGGCAACA-3′ (forward) and 5’-TGCAGTGCAGGGTCACATTC-3′ (reverse); those for CD147 were 5’-GACTGGGCCTGGTACAAGATCAC-3′ (forward) and 5′- GCCTCCATGTTCAGGTTCTCAA-3′ (reverse); and those for GADPH were 5′-TGTGTCCGTCGTGGATCTGA-3′ (forward) and 5′-CCTGCTTCACCACCTTCTTGAT-3′ (reverse).

### Western blotting

Cells were lysed in RIPA lysis buffer with protease and phosphatase inhibitors (Roche). Equal amounts of cell lysates from the protein samples were resolved by 10 and 12% SDS-PAGE and transferred onto polyvinylidene difluoride (PVDF) membranes. These membranes were incubated with 5% skimmed milk or 5% BSA in PBST at room temperature for 1 h; after rinsing 3 times, the membranes were incubated overnight at 4 °C with the following specific primary antibodies: mouse anti-human CD147 (at 1:1000, Abcam), rabbit anti-human MMP9, rabbit anti-human p38 MAPK and rabbit anti-human phosphorylated p38 MAPK (at 1:1000, Cell Signaling Technology Company). This step was then followed by incubation with horseradish peroxidase (HRP)-conjugated goat anti-mouse and anti-rabbit secondary antibodies (1:5000, Jackson) for 1 h at room temperature. The blots were visualized with an enhanced chemiluminescence kit (ECL plus). The intensity of each band was measured by using Quantity One software. The relative protein expression levels were determined by normalization to that of β-actin.

### Statistical analysis

The data were analyzed by SPSS18.0 software (Pearson; SPSS, Inc., Chicago, IL, USA) and presented as the mean ± standard deviation. The data were analyzed by Student’s t-test or one-way analysis of variance followed by T test. *P* values of less than 0.05 were considered statistically significant.

## Results

### Clinical data

Forty-two subjects were recruited for this study and divided into three groups: non-smokers (14 subjects), smokers without COPD (14 subjects) and smokers with COPD (14 subjects). The primary clinical and lung function data for these subjects in the study are listed in Table 1. In the smokers with COPD group, 12 subjects were GOLD 2 stage, and 2 were GOLD 3 stage. There was no difference in age or sex among all subjects. Cigarette smoke history was not significantly different between smokers without COPD and smokers with COPD (*p* > 0.05). The number of smoking years was higher in smokers with COPD than in smokers without COPD (*p* < 0.05). FEV1 (% predicted) and the FEV1/FVC ratio were significantly lower in smokers with COPD than in non-smokers and smokers without COPD (*p* < 0.05).

**Table 1:** Clinical and lung function characteristics of all patients

	Non-smokers	Smokers without COPD	Smokers with COPD
Subject (n)	14	14	14
Age (years)	61.5 ± 4.7	60.7 ± 5.0	62.1 ± 3.9
Sex (male/female)	10/4	13/1	11/3
Smoking history (pack-years)	0	52.8 ± 8.6	60.3 ± 7.4^a^
Smoking years	0	26.9 ± 1.5	38.8 ± 0.8^b^
FEV1 (% predicted)	89.8 ± 3.8	87.3 ± 4.1	60 ± 5.8^c^
FEV1/FVC (%)	85.3 ± 5.9	83 ± 4.9	62.8 ± 5.2^d^
GOLD stage			
1	–	–	0
2	–	–	12
3	–	–	2
4	–	–	0

The data are presented as the mean (s.d.). ^a^*p* > 0.05 vs smokers without COPD, ^b, c, d^*p* < 0.05 vs smokers without COPD

### HE and AB-PAS airway staining

**Fig. 1:**
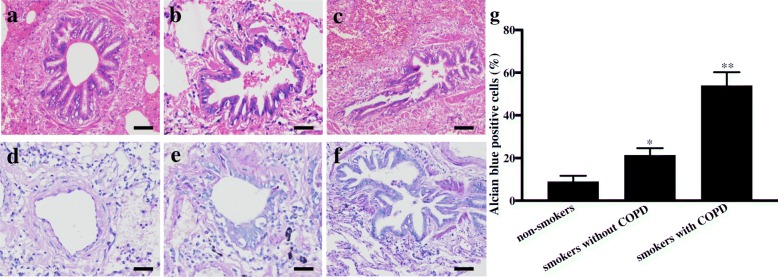
Inflammation response and mucus secretion in the lung specimens. Representative photomicrographs of HE staining in airways from non-smokers without COPD (**a**), smokers without COPD (**b**) and smokers with COPD (**c**). Representative photomicrographs of AB-PAS staining from non-smokers without COPD (**d**), smokers without COPD (**e**) and smokers with COPD (**f**). Scale bars = 50 μm. Percentage of PAS-positive cells in all the airways in the three groups (**g**). *p < 0.05, compared with the non-smokers group, **p < 0.05, compared with the smokers without COPD group

To determine the inflammation response and mucus secretion in the lung specimens, HE was used to stain areas with inflammation, and AB-PAS was used to stain mucin glycoproteins. The HE staining results revealed that the airway epithelial cells in smokers with COPD had epithelial integrity destruction and inflammatory cell infiltration. The airway epithelial cells in non-smokers without COPD were well-arranged, with complete basement membranes and less inflammatory cell infiltration, and the pathological features of the cells in smokers without COPD were in between those in non-smokers and smokers with COPD (Fig. 1 a, b, c). There were 54% AB-PAS-positive areas in the smokers with COPD group; the non-smokers without COPD group had 5% AB-PAS-positive areas, and the percentage of AB-PAS-positive areas in the smokers without COPD group (21.3%) was in between them (Fig. 1 d, e, f, g).

### Expression of MUC5AC and CD147 in lung specimens

**Fig. 2:**
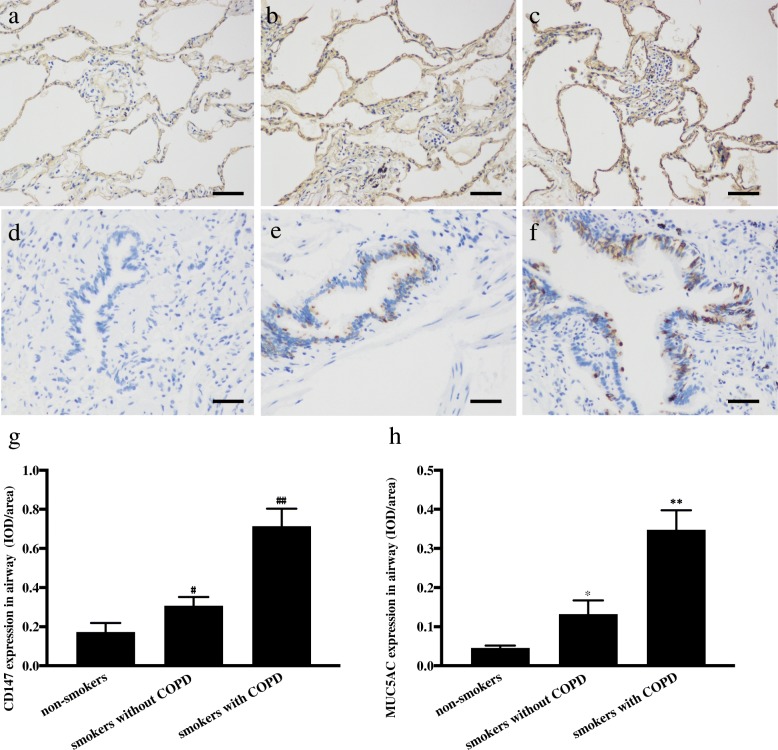
CD147 and MUC5AC expression in airways in the lung specimens. Photomicrographs of brown staining show the expression of CD147 in non-smokers (**a**), smokers without COPD (**b**) and smokers with COPD (**c**). Photomicrographs of brown staining show MUC5AC secretion in non-smokers (**d**), smokers without COPD (**e**) and smokers with COPD (**f**). Scale bars = 50 μm. Integrated optical density (IOD)/area represents the expression of CD147 (**g**) and MUC5AC (**h**). ^#^*p* < 0.05, compared with non-smokers, ^##^*p* < 0.05, compared with smokers without COPD. ^*^p < 0.05, compared with non-smokers, ^**^p < 0.05, compared with smokers without COPD

The average optical density was used to determine the expression of CD147 and MUC5AC. IHC demonstrated that the average optical density of CD147 in smokers without COPD was more than two-fold that in smokers without COPD (*p* < 0.05); there was more CD147 expression in smokers without COPD than in non-smokers without COPD (*p* < 0.05). The average optical density of MUC5AC was higher in smokers with COPD than in smokers without COPD (*p* < 0.05), and the mean density in smokers without COPD was higher than that in non-smokers without COPD (*p* < 0.05) (Fig. 2). Consistent with previous data, mucin concentrations were higher in smokers with COPD than in those who had never smoked [2]. What is more, we found that CD147 was also higher in smokers with or without COPD than in non-smokers.

### Cigarette smoke extract inhibits HBE cell proliferation

**Fig. 3:**
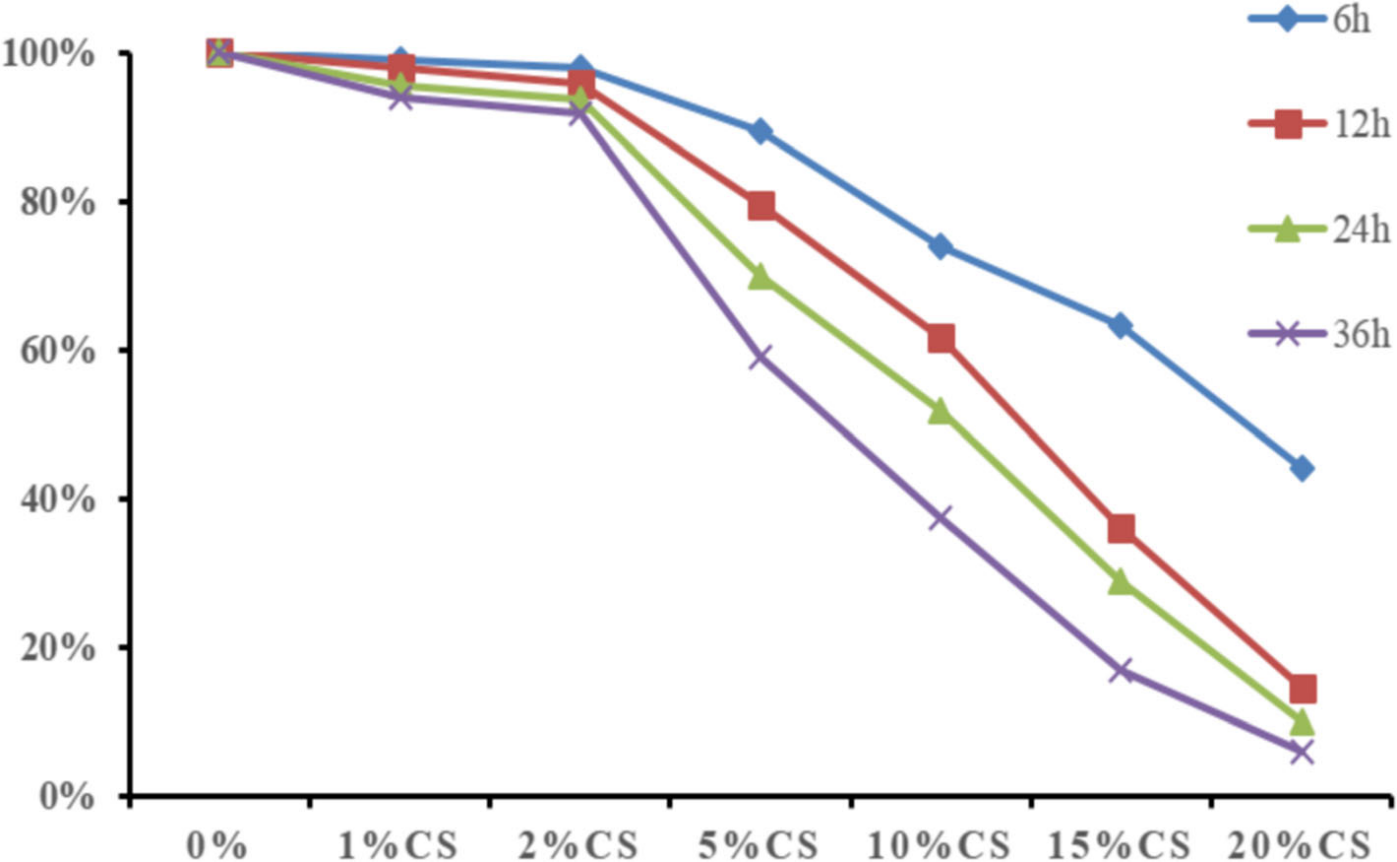
Viability of HBE cells following exposure to CS. HBE cells were exposed to 0, 1, 2, 5, 10 and 15% CS for 0, 6, 12, 24 and 36 h, and CCK8 assays were used to determine the viability of HBE cells

Cell proliferation was detected by CCK8 assay. The cytotoxic effect of CS extract on HBE cells occurred in a time- and concentration-dependent manner. Incubation with 1–2% CS extract for 6–36 h had no effect on cell proliferation, and HBE cell proliferation decreased with increasing CS extract concentrations and increasing incubation times (Fig. 3). 15% CS extract treated group has very few cells due to the high concentration of CS.

### CS extract exposure activates MUC5AC and CD147 expression in HBE cells in vitro

**Fig. 4:**
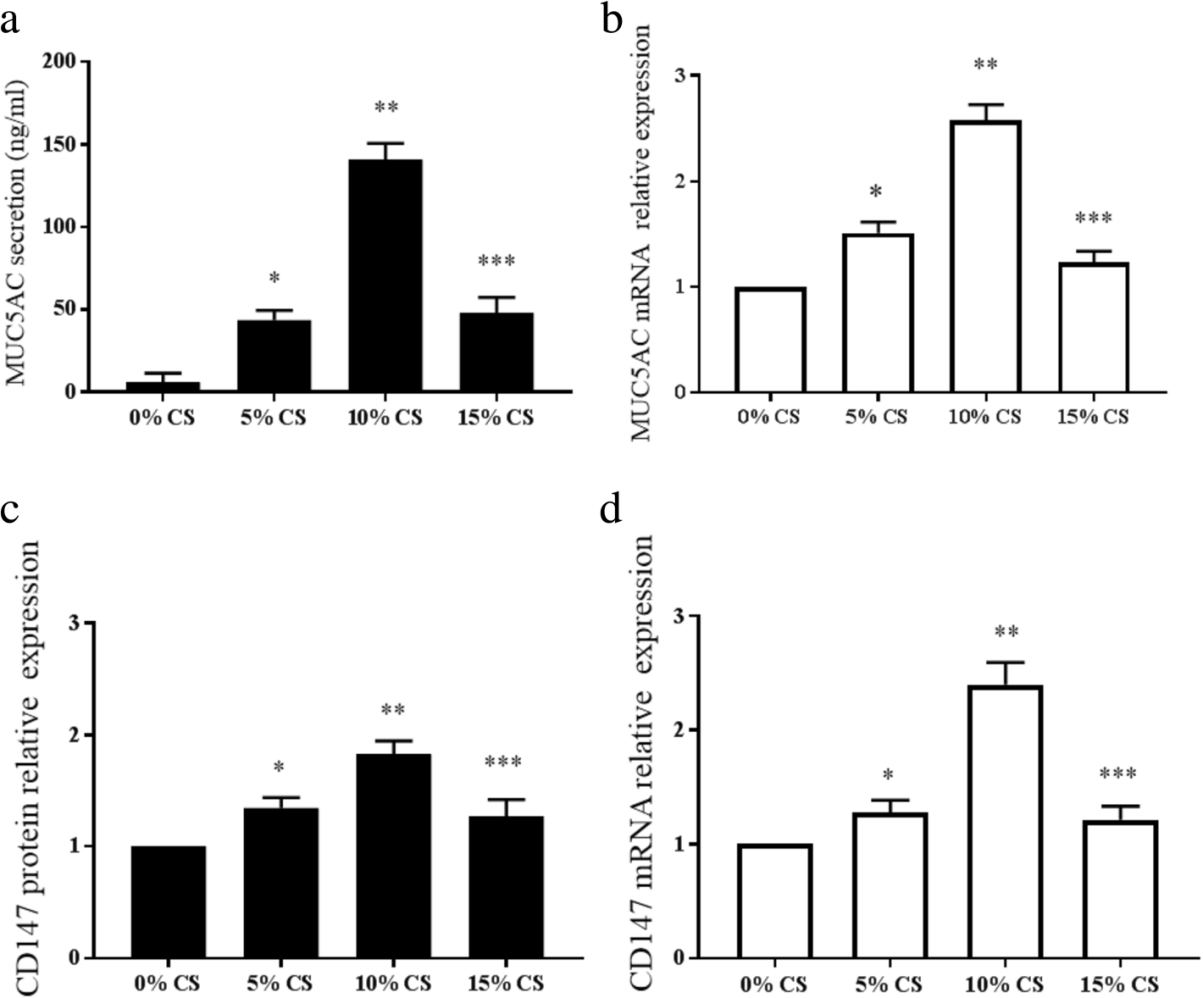
Expression of MUC5AC and CD147 induced by CS extract in HBE cells. HBE cells were treated with various concentration of CS extract (from 5 to 15%) for 24 h. MUC5AC protein (**a**) and mRNA (**b**) levels were detected by ELISA and RT-PCR, respectively. CD147 protein (**c**) and mRNA (**d**) levels were assessed by Western blotting and RT-PCR, respectively. ^*^p < 0.05, compared with 0% CS extract, ^**^p < 0.05 vs. compared with 5% CS extract, ^***^p < 0.05, compared with 10% CS extract. The data are representative of 3 separate experiments

HBE cells were treated with different concentration of CS extract for 24 h. CS extract (0–10%) induced MUC5AC and CD147 expression in a concentration-dependent manner at the mRNA and protein levels after 24 h of incubation. The mRNA expression level of MUC5AC was increased by approximately 1.5- and 2.6-fold following incubation with 5 and 10% CS, respectively (*p* < 0.05). Stimulation with 0, 5 and 10% CS induced averages of 6 ng/ml, 43 ng/ml and 161 ng/ml MUC5AC protein secretion in the culture supernatant (Fig. 4). The mRNA expression of CD147 was increased by approximately 1.3- and 24-fold after 24 h of incubation with 5 and 10% CS (*p* < 0.05). Stimulation with 5 and 10% CS induced 1.3- and 1.8-fold increases in CD147 protein expression (*p* < 0.05) (Fig. 4). However, the expression of MUC5AC and CD147 in HBE cells was lower after treatment with 15% CS extract than after treatment with 10% CS extract (Fig. 4). As shown in Fig. 3, cell viability was highly affected because the cell number decreased. These data suggested that in vitro CS extract induces MUC5AC and CD147 production, which was in agreement with the lung specimen results.

### CD147 deficiency impairs CS-induced MUC5AC secretion in HBE cells

**Fig. 5:**
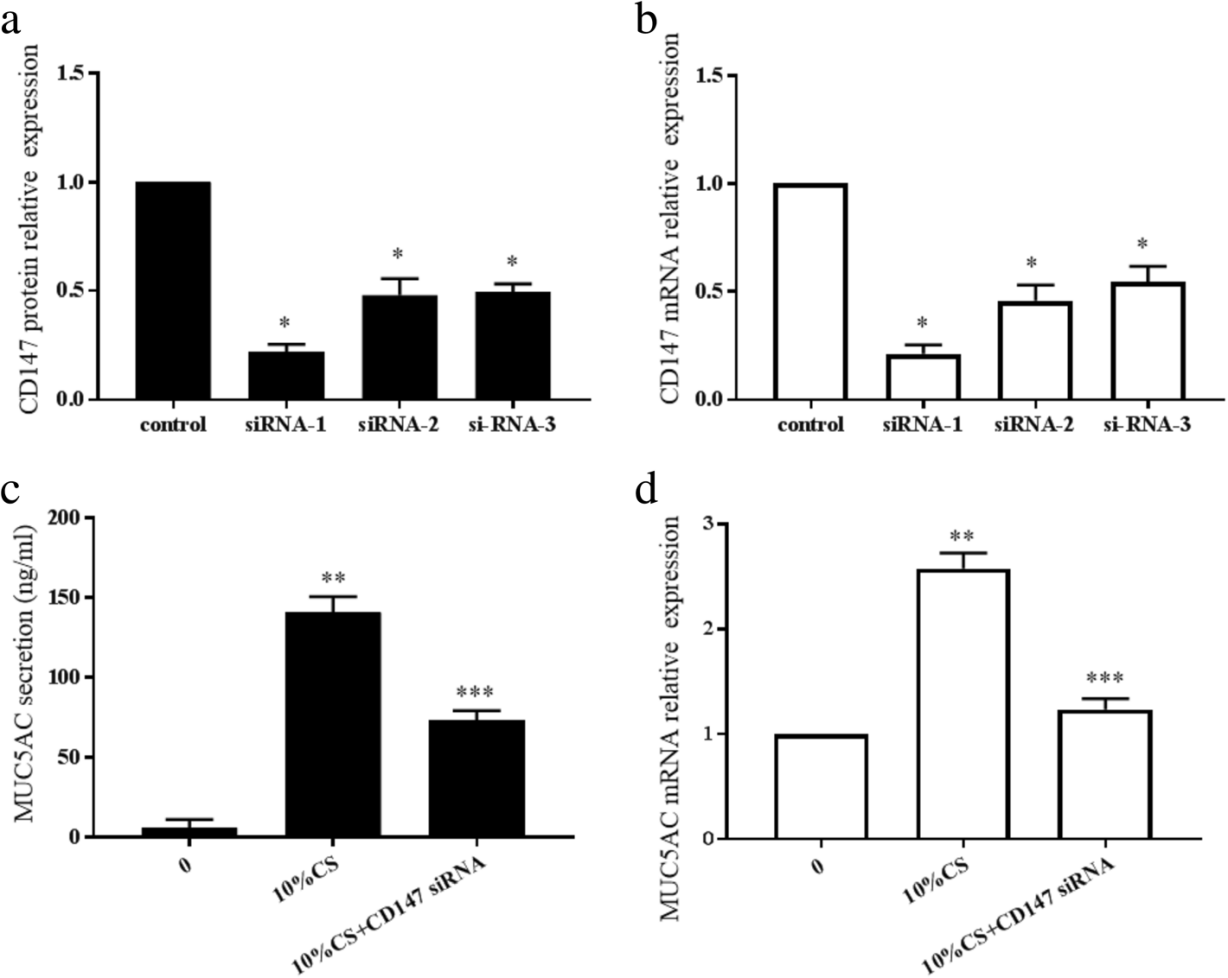
Changes in MUC5AC after CD147 silencing by siRNA transfection. CD147 protein (**a**) and mRNA (**b**) expression levels were assessed after siRNA transfection. Three CD147 siRNA were designed, and CD147 siRNA 1 was the most effective at silencing CD147 expression. *p < 0.05, compared with control transfection. MUC5AC secretion (**c**) and MUC5AC mRNA expression (**d**) were detected after CD147 silencing. **p < 0.05, compared with the control group, ***p < 0.05, compared with the 10% CS extract group. The results are representative of 3 independent experiments

To functionally connect CD147 to MUC5AC secretion, we depleted CD147 using small interfering RNA transduced with Lipofectamine 3000. We established three CD147 siRNAs to transfect HBE cells; all siRNA silenced CD147 expression, and the one inducing the greatest silencing was chosen for the subsequent experiments (Fig. 5). HBE cells were transfected with CD147 siRNA and then incubated with 10% CS extract for 24 h. CD147 knockdown by siRNA resulted in an approximately 1-fold reduction in the MUC5AC mRNA and protein expression levels compared to siRNA control treatment (*p* < 0.05) (Fig. 5).

### CD147 regulates CS-induced MUC5AC production through the MMP9/p38 MAPK pathway

To further explore how CD147 regulates MUC5AC production, we used signaling molecule inhibitors to search for the possible signaling pathway. HBE cells were pretreated with 10 μM SB-3CT (MMP9 inhibitor) or 10 μM SB203580 (p38 MAPK inhibitor) for 1 h prior to CS extract incubation.

**Fig. 6:**
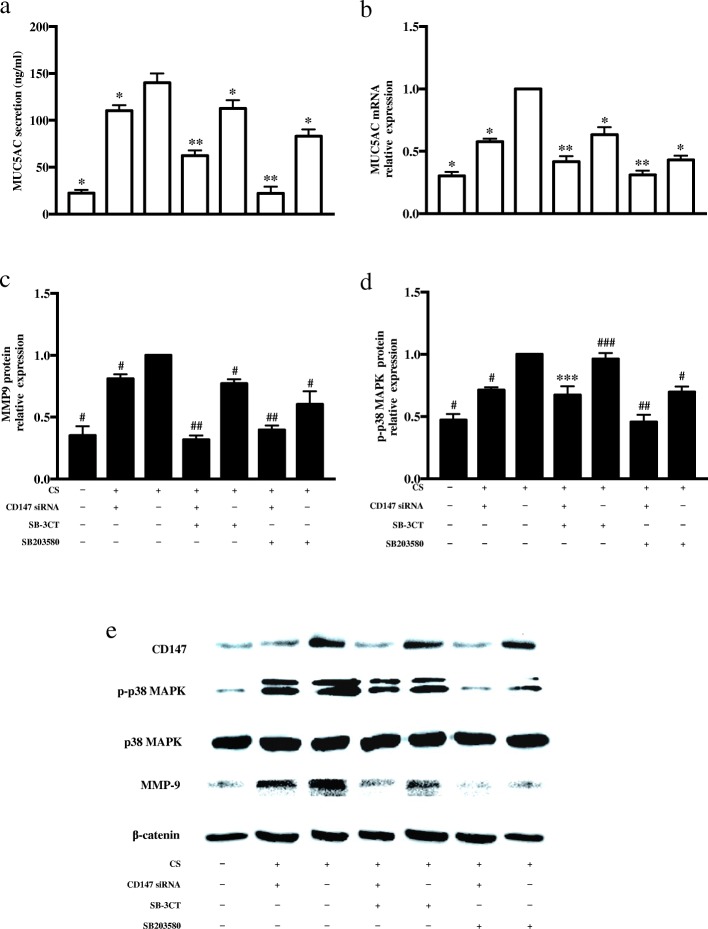
CD147 regulates the p38 MAPK/MMP9 signal pathway and is involved in CS-induced MUC5AC secretion in HBE cells. Cells were transfected with CD147 siRNA and pretreated with 10 μM SB203580 (p38 MAPK inhibitor) or 400 nM SB-3CT (MMP9 inhibitor) for 1 h; then, the cells were stimulated with 10% CS. After 24 h, MUC5AC protein (**a**) and mRNA (**b**) levels were detected. *p < 0.05, compared with the CS extract treatment group, **p < 0.05, compared with the CS + CD147 siRNA treatment group. Cell extracts were subjected to Western blotting analysis to detect MMP, p-p38 MAPK and total p38 MAPK (**c**, **d** and **e**). ^#^*p* < 0.05, compared with the CS extract treatment group, ^##^*p* < 0.05, compared with the CS + CD147 siRNA treatment group, ***p > 0.05, compared with the CS+ CD147 siRNA treatment group, ^###^*p* > 0.05 compared with the CS extract treatment group

Pre-treatment with SB-3CT or SB203580 markedly abrogated the increase in MUC5AC mRNA expression and protein production induced by CS (*p* < 0.05 vs. untreated group) (Fig. 6).

HBE cell exposure to CS led to significant increases in the levels of MMP9 and phosphorylated p38 MAPK (p-p38 MAPK) (*p* < 0.05 vs. untreated group); MMP9 expression and p-p38 MAPK were markedly inhibited by SB203580 (*p* < 0.05 vs. CS-treated group), and SB-3CT attenuated MMP9 expression (*p* < 0.05 vs. CS treated group) but did not affect p-p38 MAPK (*p* > 0.05 vs. CS-treated group) (Fig. 6). In this cell model, we verified that CS induced MUC5AC expression through the p38 MAPK/MMP9 signaling pathway.

Western blot analysis revealed that transfection with CD147 siRNA prior to incubation with CS markedly reduced MUC5AC secretion and MMP9 and p38 MAPK expression (*p* < 0.05, vs. CS-treated group), and pretreatment with SB-3CT or SB203580 caused additional reductions in MMP9 and p-p38 MAPK (*p* < 0.05, vs. CD147 siRNA + CS treatment group) (Fig. 6).

Considering the non-specific inhibition of SB-3CT and SB203580, we also detected the expression of MMP2, which can be inhibited by SB-3CT, and the expression of phosphorylated Akt, which can be inhibited by SB203580; we found that although CS extract increased the expression of MMP2 and phosphorylation of Akt, CD147 had no effect on their expression (Additional file 1: Figure S1). Together, these data suggested that CD147 induced MUC5AC via the p38 MAPK/MMP9 signaling pathway.

## Discussion

CD147 is a transmembrane glycoprotein that is broadly expressed in several types of epithelial cells [15]. Gwin et al. found that CD147 was increased in the airways of asthmatic mice, and in vivo treatment with an anti-CD147 mAb significantly reduced the accumulation of eosinophils and airway epithelial mucin production [23]. Blemoycin-induced lung injury was associated with increased CD147 expression in the bronchiolar epithelium and BALF in a study by Betsuyaku et al. [19]. These studies have clearly demonstrated that CD147 is involved in lung inflammation responses [24]. In our study, COPD lung specimens were utilized to determine the expression of CD147 and MUC5AC. We discovered that CD147 and MUC5AC were more highly expressed in smokers with COPD than in smokers without COPD, and their expression levels were also higher in smokers than in non-smokers. In vitro CS extract incubation increased CD147 production and MUC5AC secretion in human bronchial epithelial cells, which was in agreement with the results from lung specimens. Together, our data strongly implicated that CD147 was increased in lung inflammatory diseases.

Cigarette smoke is a primary cause of mucus secretion, and a variety of signaling molecules participate in the regulation of MUC5AC production induced by CS extract [9, 10, 12]. Hitesh et al. found that increased MUC5AC transcription and protein levels were associated with increased lung MMP9 levels in acrolein-exposed mice [25]. Hyung et al. demonstrated that insulin induces MUC5AC expression via the p38 MAPK signaling pathway in human airway epithelial cells [26]. Based on these studies, we utilized an MMP9 inhibitor (SB-3CT) and a p38 MAPK inhibitor (SB203580) to pretreat HBE cells and then stimulated them with CS extract; in accordance with previous studies, we found that inhibiting MMP9 and p38 MAPK attenuated MUC5AC production.

It is well known that CD147 is responsible for stimulating the secretion and activation of MMPs, which is associated with inflammatory damage [14, 18]. In addition to MMPs, a series of studies has shown that CD147 regulates different subclasses of the MAPK superfamily in different bioprocesses and cell lines [16, 27]. Melissa et al. reported that p38 MAPK was required for the CD147 stimulation of MMP in stromal fibroblasts in lung cancer [28]. Oppositely, another study showed that after cyclophilin A stimulation, CD147-induced inflammatory activation could not be blocked by a p38 MAPK inhibitor in a human macrophage-like cell line, THP-1 cells [29]. These data suggest that the multiple roles of CD147 in p38 MAPK activation may be dependent on the cell types and irritants. In our study, we used small interfering RNA to reduce CD147 expression to determine whether CD147 also regulated MMP9 and p38 MAPK production. Interestingly, our work shows that CD147 deficiency significantly reduced MUC5AC secretion and MMP and phosphorylated p38 MAPK production. Further studies are required to determine the exact mechanisms of the different functions of CD147 in different cells.

## Conclusions

In summary, we proved that CD147 is more highly expressed in the lung specimens of smokers with COPD than in those of control subjects. In vitro, CD147 plays a promoting role in cigarette smoke-induced MUC5AC secretion, and the p38 MAPK/MMP9 signaling pathway is involved in this inducing regulation. In addition, considering the complicated regulation network in MUC5AC secretion, future studies are required to explore whether CD147 modulates signaling molecules other than MMP9 and p38 MAPK that are involved in MUC5AC secretion. It can thus be hypothesized that CD147 deficiency protects against mucus hypersecretion in COPD, and CD147 may be a potential target for reducing mucus overproduction.

## Additional file

Additional file 1: **Figure S1.** CD147 had no effect on MMP2 and Akt phosphorylation. Cells were transfected with CD147 siRNA, then stimulated with 10% CS. After 24 h, Akt phosphorylation (**a**, **c**) and MMP2 expression (**b**, **d**) were detected. *p < 0.05, compared with the untreated group, **p > 0.05, compared with the CS-treated group. ^#^*p* < 0.05, compared with the untreated group, ^##^*p* > 0.05, compared with the CS-treated group. (PDF 113 kb)

## Abbreviations

COPD: Chronic obstructive pulmonary disease
CS extract: Cigarette smoke extract
EGFR: Epidermal growth factor receptor
ELISA: Enzyme-linked immunosorbent assay
FEV1: Forced expiratory volume in 1 s
FVC: Forced vital capacity
GOLD: Global Initiative for Chronic Obstructive Lung Disease
HBE: Human bronchial epithelial
HE: Hematoxylin-eosin
MAPK: Mitogen-induced spark protein kinase
MMP9: Matrix metalloproteinase 9
PBS: Phosphate-buffered saline
RT-PCR: Real-time polymerase chain reaction
siRNA: Small interfering RNA
