# Supplementary figures and images for: CD147 increases mucus secretion induced by cigarette smoke in COPD
Source: BMC Pulm Med. 2019 Feb 6;19:29. doi: 10.1186/s12890-019-0791-0 (PMC6364420; doi:10.1186/s12890-019-0791-0)

a

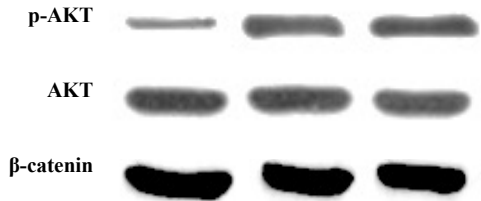

b

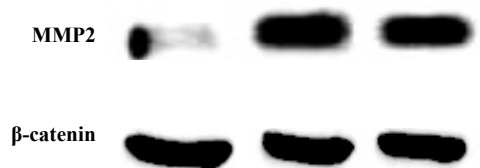

c

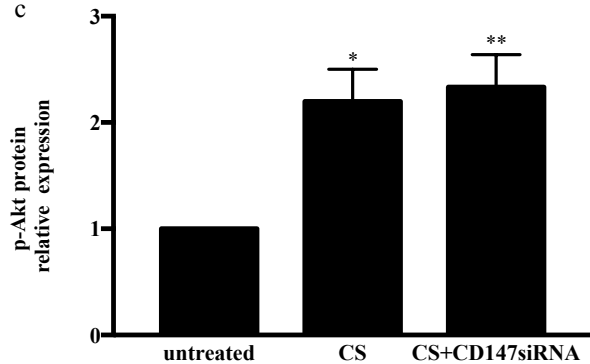

d

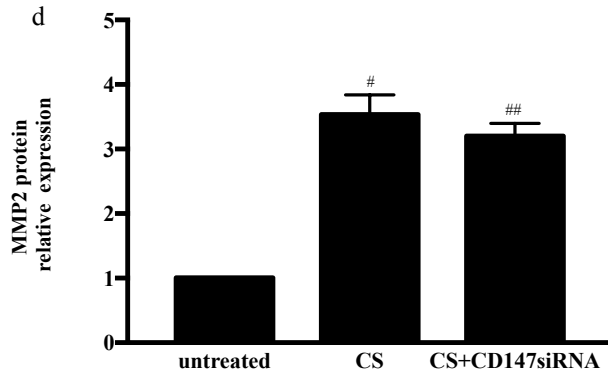

Supplement: Supplementary file 1 — Figure S1. CD147 had no effect on MMP2 and Akt phosphorylation. Cells were transfected with CD147 siRNA, then stimulated with 10% CS. After 24 h, Akt phosphorylation (a, c) and MMP2 expression (b, d) were detected. *p < 0.05, compared with the untreated group, **p > 0.05, compared with the CS-treated group. #p < 0.05, compared with the untreated group, ##p > 0.05, compared with the CS-treated group. (PDF 113 kb) [file 12890_2019_791_MOESM1_ESM.pdf]
